# Supplementary material for: Causal Relationship between Aging and Anorexia Nervosa: A White-Matter-Microstructure-Mediated Mendelian Randomization Analysis
Source: Biomedicines. 2024 Aug 16;12(8):1874. doi: 10.3390/biomedicines12081874 (PMC11351342; doi:10.3390/biomedicines12081874)
Supplement: Supplementary file 1 [file biomedicines-12-01874-s001.zip › Supplementary Figure/Figure S8/Leaveoneout plot of MR analysis for TL to ubm-b-1467.pdf]

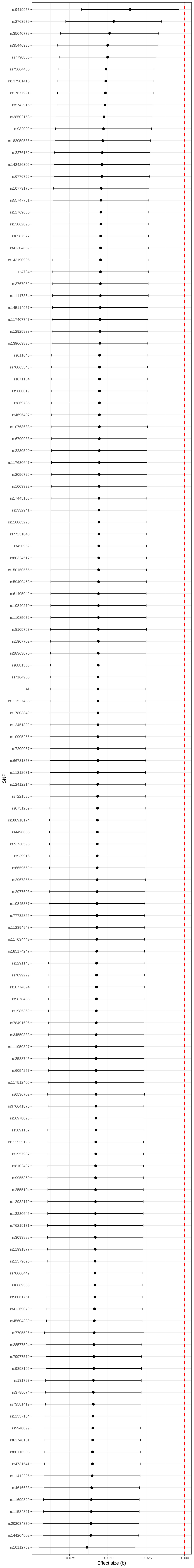
rs9419958
rs2763979
rs35640778
rs35446936
rs7790856
rs75664430
rs137901416
rs17677991
rs5742915
rs28502153
rs932002
rs182059586
rs2276182
rs142426306
rs6776756
rs10773176
rs55747751
rs11769630
rs13062095
rs6587577
rs41304832
rs143190905
rs4724
rs3767952
rs11117354
rs145114957
rs117407747
rs12925933
rs139669835
rs611646
rs76065543
rs871134
rs9600019
rs869785
rs4695407
rs10768683
rs6790988
rs2230590
rs117630647
rs2056726
rs1003322
rs17445108
rs1332941
rs116863223
rs77231040
rs450962
rs80324517
rs150150565
rs59409453
rs61405042
rs10840270
rs11085072
rs8105767
rs1907702
rs28363070
rs6881568
rs7164950
A8
rs111527438
rs17803849
rs12451892
rs10905255
rs7209057
rs66731853
rs11212631
rs12412214
rs7221585
rs6751209
rs188918174
rs4498805
rs73730598
rs939916
rs6659669
rs2967355
rs2977608
rs10845387
rs77732866
rs112394943
rs117034449
rs185174247
rs1291143
rs7099229
rs10774624
rs9878436
rs1985369
rs78491606
rs34550383
rs111950327
rs2538745
rs6054257
rs117512405
rs6536702
rs376641875
rs16978028
rs3891167
rs113525195
rs1957937
rs8102497
rs9955360
rs2555104
rs12932179
rs13230646
rs76219171
rs3093888
rs11991877
rs11579626
rs76666449
rs6669563
rs56061761
rs41269079
rs45604339
rs7705526
rs28577594
rs79977579
rs9398196
rs131797
rs3785074
rs73581419
rs11557154
rs9940099
rs61748181
rs80116508
rs4731541
rs11412296
rs4616688
rs11699829
rs11584821
rs202034370
rs144204502
rs10112752
SNP
-0.075
-0.050
-0.025
0.000
Effect size (b)
